# At what age should people with obesity start colorectal cancer screening?

**DOI:** 10.1002/ijc.70448

**Published:** 2026-04-09

**Authors:** Teresa Seum, Marko Mandic, Fatemeh Safizadeh, Michael Hoffmeister, Hermann Brenner

**Affiliations:** ^1^ Division of Clinical Epidemiology of Early Cancer Detection, German Cancer Research Center (DKFZ) Heidelberg Germany; ^2^ Medical Faculty Heidelberg Heidelberg University Heidelberg Germany; ^3^ Cancer Prevention Graduate School, German Cancer Research Center (DKFZ) Heidelberg Germany

**Keywords:** body mass index, early detection, early‐onset colorectal cancer, screening

## Abstract

The incidence of obesity and early‐onset colorectal cancer (CRC) has rapidly increased in recent decades. We estimated CRC risk differences by body mass index (BMI) and derived corresponding BMI‐specific CRC screening initiation ages across multiple countries, to help refine the timing of CRC prevention. We based our analyses on population‐level CRC incidence data from the GLOBOCAN database (2022) and risk estimates from a meta‐analysis of six cohort studies. The analysis included adults aged 30 to 60 years from six countries: United States, Canada, Germany, France, United Kingdom, and Italy. Individuals with overweight were used as the reference category, reflecting the average BMI of adults eligible for screening in these countries. Risk‐adapted screening initiation ages were defined as the ages at which individuals with obesity reached the same 5‐year cumulative CRC risk as those with overweight at ages 45 and 50 (aCR_45_ and aCR_50_). Compared with individuals with overweight, those with normal weight had a lower risk of early‐onset CRC (OR, 0.76; 95% CI, 0.68–0.84), while those with obesity had higher risk (OR, 1.42; 95% CI, 1.04–1.95). Across countries, individuals with obesity reached the aCR_45_ at age 41 to 42 years and the aCR_50_ at age 46 to 47 years. These findings indicate that individuals with obesity reached CRC screening thresholds up to 3–4 years earlier than those at average risk. BMI may offer a practical and scalable marker to inform personalized CRC screening initiation, with the potential to enhance early detection if integrated into existing screening programs.

## Graphical abstract

What's new?

Early‐onset colorectal cancer (CRC) incidence is rising in the United States, Canada, and parts of Europe, even as rates in older adults stabilize or decline. Excess body weight, which is a known CRC risk factor, is a potential contributor to this trend. The present study evaluated CRC screening initiation ages and whether adjustments should be made according to body mass index (BMI). Analyses show that relative to adults with overweight, individuals with obesity reach the same cumulative CRC risk levels 3–4 years earlier. The findings lend support to the use of BMI‐adjusted screening recommendations to enhance CRC prevention.

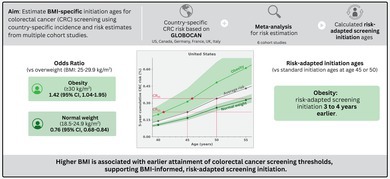

**Abbreviations**
aCR: cumulative 5‐year CRC risk for the average‐risk population
BMI: body mass index
CRC: colorectal cancer
eoCRC: early‐onset colorectal cancer
GLOBOCAN: Global Cancer Observatory
OR: odds ratio

## 1 INTRODUCTION

The incidence of early‐onset colorectal cancer (eoCRC) is increasing in the United States, Canada, and many European countries, in contrast to stabilizing or declining overall colorectal cancer (CRC) incidence in older adults.^1^, ^2^, ^3^ In the past two decades, CRC incidence under the age of 50 has been rising from 5.9 to 8.4 per 100,000 in the United States and increased up to threefold in some European countries.^1^, ^4^ As current CRC screening programs primarily target older populations, this shift in incidence highlights a potential gap in early detection strategies.

Although the etiological factors underlying the rise in eoCRC remain incompletely understood, evidence suggests that excess body weight is a key contributor.^5^ Obesity is associated with chronic low‐grade inflammation, insulin resistance, and gut microbiome alterations, all of which may contribute to colorectal carcinogenesis.^6^ Recent genomic studies also indicate that obesity‐related metabolic and inflammatory conditions could affect tumor development by influencing the genomic context in which driver mutations arise.^7^, ^8^ In the United States and most European countries, obesity prevalence has increased substantially in recent decades, with projections indicating continued growth, particularly among younger generations.^9^, ^10^ Excess body weight is estimated to account for approximately 16.2% of colon cancer cases in the United States, underscoring its substantial role in disease burden.^11^ This growing burden suggests that obesity may be an important driver of rising eoCRC incidence,^12^ supporting the need to reassess CRC screening recommendations for individuals with obesity.

Current screening recommendations are primarily age‐based, with initiation typically at age 45 in the United States and at age 50 in Canada and in most European countries, including Germany, France, the United Kingdom, and Italy.^13^, ^14^, ^15^, ^16^ This uniform approach may result in delayed detection among younger individuals at higher risk due to excess weight. While CRC risk is influenced by multiple genetic and lifestyle factors, most of these are not systematically captured in population‐level data or routinely available for screening stratification.^17^, ^18^ In contrast, body mass index (BMI) is widely recorded, consistently associated with eoCRC across diverse study designs, and can be feasibly integrated into existing programs.^19^, ^20^ These characteristics make BMI a practical starting point for exploring how risk varies across the population in ways that may inform screening programs, while acknowledging that additional risk factors could further refine individual‐level recommendations.

Therefore, this study aims to determine risk‐adapted starting ages for CRC screening for individuals with obesity. We applied a novel approach assuming that population‐level CRC risk estimates reflect the risk of individuals who are overweight, as the average BMI in North American and European populations at the most commonly recommended starting ages of screening (45 or 50) typically falls within the overweight range (25–29.9 kg/m^2^).^21^ Using this reference, we identified the ages at which individuals with obesity reach the same cumulative CRC risk as those with overweight at the screening initiation ages of 45 and 50 years.

## 2 METHODS

To calculate risk‐adapted screening ages, we combined data from a meta‐analysis on the association between excess weight and eoCRC risk with population‐based cumulative CRC risk estimates from the six populous countries in North America and Europe.

Firstly, we extracted data to evaluate the eoCRC risk associated with excess weight from the most recent and comprehensive meta‐analysis. The meta‐analysis was identified through a systematic literature search (outlined in the Data S1, Methods), which resulted in 13 meta‐analyses assessing the association between BMI and CRC risk. For the analysis, priority was given to meta‐analyses that (1) reported risk estimates specifically for eoCRC and (2) explicitly addressed potential bias due to cancer‐associated pre‐diagnostic weight loss,^22^ a common phenomenon in CRC patients that can lead to underestimation or reversal of the BMI–CRC association.^23^, ^24^ Only one meta‐analysis met both criteria and was therefore selected. Among the remaining studies, most focused on CRC overall and did not account for pre‐diagnostic weight loss, which has rarely been considered in previous studies.^20^

Secondly, we retrieved population‐based data on the cumulative risk of CRC (ICD‐10 codes C18‐C21) from the GLOBOCAN 2022 database (https://gco.iarc.fr/today/en). GLOBOCAN provides standardized, country‐level cancer incidence estimates derived from population‐based cancer registry data and validated modeling, enabling consistent comparisons across countries. We extracted 5‐year cumulative risks at ages 30, 35, 40, 45, 50, and 55 for the United States and Canada, and for Germany, France, the United Kingdom, and Italy. The analysis was restricted to individuals aged 30 to 60 years to capture the period during which early‐onset CRC risk begins to increase and to align with current screening initiation ages. A 5‐year cumulative risk timeline was chosen to reflect short‐term screening‐relevant risk and to allow a comparison with guideline‐based screening thresholds. The four European countries were selected because they have large populations, established CRC screening programs, and high‐quality registry data available through GLOBOCAN. Because the focus was on age groups younger than the standard initiation age for screening in these countries, these risk estimates largely reflect unscreened populations. Detailed descriptions of the data sources for the included countries are provided in Table S1.

We applied linear interpolation to estimate eoCRC risks at single‐year age increments between 30 and 60, using population‐based 5‐year cumulative risk data. Given that the average adult BMI at those ages in the included countries falls within the overweight category [mean BMI in Europe: 26.4 kg/m^2^, in Americas: 27.6 kg/m^2^],^21^ we considered the provided population‐level risk as representative of individuals with overweight, as this category reflects the mean BMI in these populations and offers a more stable baseline than the less prevalent normal‐weight group. Risk curves for individuals with normal weight and obesity were adjusted by applying log‐transformed odds ratios and combining variances. Estimates were then transformed to obtain adjusted odds ratios and 95% confidence intervals. BMI was categorized according to World Health Organization definitions as normal weight (18.5–24.9 kg/m^2^), overweight (25.0–29.9 kg/m^2^), and obesity (≥30.0 kg/m^2^).

Risk‐adapted screening ages were identified by determining the ages at which individuals with obesity reached the 5‐year cumulative CRC risk of individuals with overweight at ages 45 (aCR_45_) and 50 (aCR_50_), the most common screening initiation ages in the analyzed countries. As a sensitivity analysis, we repeated the analysis by using individuals with normal weight as the reference risk level.

All analyses were conducted using Python 3.11.7.

## 3 RESULTS

As described in detail in the Data S1 (Methods), the meta‐analysis published by Li et al.^19^ provided pooled risk estimates for eoCRC in individuals with overweight (BMI 25–29.9 kg/m^2^) and obesity (BMI ≥30 kg/m^2^), and the included studies contributing to these pooled estimates generally controlled for the major demographic, lifestyle, and clinical factors known to influence CRC risk. The findings of this meta‐analysis showed that individuals with overweight had an odds ratio (OR [95% confidence intervals (CI)]) of 1.32 (95% CI, 1.19–1.47) for eoCRC compared to those with normal weight (18.5–24.9 kg/m^2^), while those with obesity had an OR of 1.88 (95% CI, 1.40–2.54).^19^ These recalculated OR were 0.76 (95% CI, 0.68–0.84) and 1.42 (95% CI, 1.04–1.95) for people with normal weight and people with obesity, respectively, compared to those with overweight.

Table S2 presents the 5‐year cumulative risk of CRC from age 30 to 60. The data demonstrated a consistent increase in CRC risk from ages 30 to 60, with the highest risks observed in the United States, followed by Canada and France, while Germany showed the lowest risk levels.^25^

### 3.1 Risk‐adapted starting ages

**FIGURE 1:**
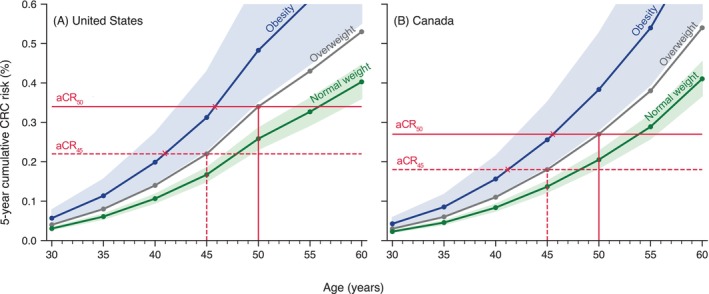
Country‐specific derivation of risk‐adapted starting ages of colorectal cancer screening based on body mass index in the United States (A) and Canada (B). CRC, colorectal cancer; aCR_45/50_ cumulative 5‐year CRC risk at age 45/50 for the average‐risk population.

**FIGURE 2:**
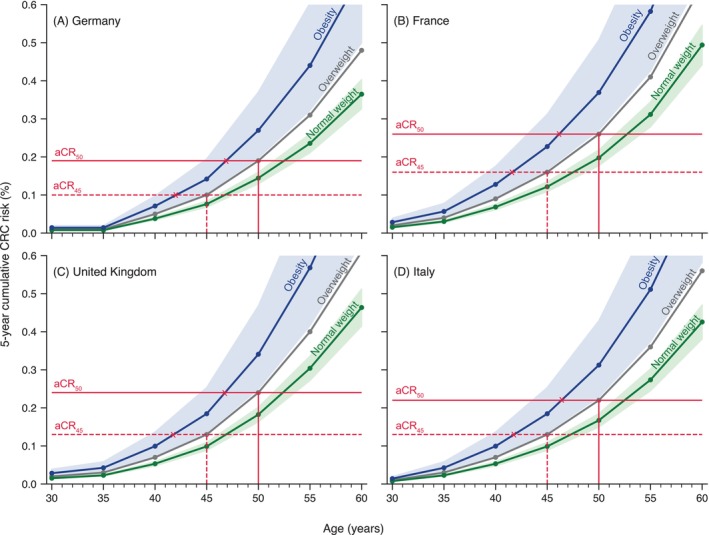
Country‐specific derivation of risk‐adapted starting ages of colorectal cancer screening based on body mass index in Germany (A), France (B), the United Kingdom (C), and Italy (D). CRC, colorectal cancer; aCR_45/50_ cumulative 5‐year CRC risk at age 45/50 for the average‐risk population.

As shown in Table 1, this analysis uses the GLOBOCAN cumulative CRC risk as a representation of the average‐risk population, representing individuals with overweight in the included countries. Individuals with obesity reached the aCR_45_ benchmark risk levels at age 41 (95% CIs, 38–45) in the United States and Canada (Figure 1), and at age 42 in the included European countries (Figure 2). For the aCR_50_ benchmark, individuals with obesity reached equivalent risk levels at age 46 (95% CI, 42–49) in the United States and Canada, while the risk‐adapted screening age was 47 years (95% CIs, 44–50) in Germany and the United Kingdom, and 46 years (95% CIs, 43–50) in France and Italy.

**TABLE 1:** Risk‐adapted starting ages for colorectal cancer screening for individuals with obesity (BMI ≥30 kg/m^2^), by country.

	Risk‐adapted starting age [years (95% confidence interval)]	
	Benchmark: Age 45	Benchmark: Age 50
North America		
United States	41 (38–44)	46 (42–49)
Canada	41 (38–45)	46 (42–49)
Europe		
Germany	42 (40–45)	47 (45–50)
France	42 (39–45)	46 (43–50)
United Kingdom	42 (40–45)	47 (44–50)
Italy	42 (40–45)	46 (44–50)

*Note*: Main analysis using people with overweight (BMI 25–29.9 kg/m^2^) as the reference category.

Abbreviation: BMI, body mass index.

In the sensitivity analyses shown in Table S3, it was assumed that the GLOBOCAN cumulative CRC risk reflects the risk of individuals with normal weight. Based on this assumption, risk‐adapted screening ages for individuals with overweight, using the aCR_45_ benchmark, were 42 years (95% CI, 41–43) in the United States, Canada, the United Kingdom, Italy, and France, and 43 years (95% CI, 42–43) in Germany. Individuals with obesity reached equivalent risk levels at ages 38 to 40 across countries, with the earliest in the United States (age 38; 95% CI, 36–41) and the latest in the selected European countries (age 40; 95% CI, 37–42). For the aCR_50_ benchmark, individuals with overweight reached equivalent risk levels at 46 to 47 years across all countries. Among individuals with obesity, risk‐adapted screening ages ranged from 42 years in Canada (95% CI, 40–46) to 45 years in Germany and the United Kingdom (95% CI, 42–47).

## 4 DISCUSSION

The significant rise in eoCRC in Europe and North America underscores the need for tailored screening strategies to improve early detection and outcomes. Our findings show that individuals with obesity reach the same cumulative CRC risk level as those with overweight 3–4 years earlier across all included countries. These results support the recommendation of BMI‐adjusted screening recommendations to enhance CRC prevention in higher‐risk individuals.

To the best of our knowledge, this is the first study to quantify BMI‐specific screening initiation ages for CRC. As a widely available and easily measurable risk factor, BMI could be feasibly integrated into clinical and public health screening programs without the need for specific laboratory measures, such as genetic testing, or for comprehensive risk factor ascertainment.^26^, ^27^ Nevertheless, CRC risk is multifactorial, and BMI represents only one risk factor among several established determinants, including genetic and lifestyle factors, family history, and other medical conditions.^17^ Accordingly, phenotypic models including additional risk factors may further enhance risk discrimination and the efficiency of CRC screening, particularly in settings where such data are routinely available.^28^ Thus, BMI‐based initiation ages should be viewed as a pragmatic starting point, particularly for population‐level implementation, rather than a complete representation of CRC risk.

A recent modelling study from the United States suggested that starting CRC screening earlier among people with obesity may be cost‐effective, and that it remains to be decided whether BMI should be used as a single predictor or incorporated into a multivariable tool to tailor CRC screening.^29^ Our estimates help inform both approaches by quantifying the magnitude of BMI‐associated shifts in screening‐relevant risk thresholds.

The association between CRC and excess weight is well‐established, as due to obesity chronic inflammation, insulin resistance, and adipokine imbalances can create a tumor‐promoting microenvironment.^6^ Additionally, emerging evidence also suggests gut microbiota dysbiosis as an additional factor.^30^ The significance of obesity is underscored by the substantial burden and growing prevalence. For example, in the United States, the current prevalence of obesity among adults aged 40 to 59 is 46.4%, which is the highest among the age groups. Projections indicate that obesity rates will continue to increase in the United States and also in Europe, reaching peak levels between 2026 and 2054.^31^ While the average BMI at screening age remains in the overweight range in most of the countries included in this study, recent data suggest that mean BMI in the United States has reached levels consistent with obesity.^32^ The use of the overweight category as the reference group was intended to represent an average screening‐eligible risk profile rather than the exact population mean in each country. In settings such as the United States, where mean BMI is higher, the estimated risk‐adapted screening initiation ages may therefore be conservative. This shift aligns with the country's recent change in CRC screening guidelines, lowering the recommended screening initiation age from 50 to 45 years.^33^

Although the age‐specific risk curves derived in our main analysis might also suggest potential postponement of starting ages above 45 and 50, they should not be used for that purpose because the increase of risk at higher ages is largely affected and attenuated by already established screening programs. This particularly applies to the United States where widespread screening colonoscopy uptake has strongly reduced CRC incidence at higher ages in the past decades.^34^

The successful implementation of risk‐adapted CRC screening requires addressing disparities that prevent individuals with obesity from accessing preventive care. Individuals with obesity often face barriers to CRC screening, including stigma, logistical challenges, and negative healthcare experiences.^35^ BMI‐based screening approaches may also face challenges related to social acceptance and risk communication, particularly in settings where higher body weight is common. Educational initiatives, training to mitigate weight bias, and ensuring accessible medical equipment are essential for reducing these disparities.^36^ Framing BMI as a risk indicator rather than a moral judgment could help mitigate these concerns. By promoting an inclusive healthcare environment, such measures may increase screening adherence and equity.^35^, ^36^

This study has several key strengths, including the use of high‐quality, up‐to‐date cancer incidence data and the consideration of excess weight related risk estimates accounting for potential bias from pre‐diagnostic weight loss.^37^ However, several limitations should be acknowledged. First, the analysis focuses on a limited number of predominantly high‐income Western countries with specific BMI distributions and CRC epidemiology and the assumption of two uniform starting ages for average‐risk populations across different regions. These characteristics may restrict the generalizability of our findings, although the methodology is adaptable to settings with different screening policies, demographic profiles, or BMI distributions. Second, while BMI is a widely used and practical measure of adiposity, it does not differentiate between muscle and fat mass or account for fat distribution, making it less precise than indicators of central obesity like waist circumference or waist‐to‐hip ratio.^38^ BMI cut‐offs also vary between countries and regions,^39^ which may influence both category prevalence and associated risk estimates; therefore, the applicability of our findings is limited to the populations and BMI definitions analyzed in this study. Nevertheless, at the population level, BMI remains a strong predictor of overall body fatness and an accessible tool for risk stratification in screening programs. Finally, translating pooled risk estimates into risk‐adapted ages assumes stability of BMI‐related associations across demographic subgroups; although the underlying studies generally controlled for major confounders, residual confounding cannot be ruled out.

In conclusion, our findings may help inform implementation of risk‐adapted CRC screening by providing a straightforward approach to define risk‐adapted starting ages for individuals with obesity. As obesity prevalence and eoCRC incidence continue to rise, tailored screening strategies could become increasingly relevant for limiting the burden of eoCRC. While refined risk stratification can improve efficiency, it may also introduce challenges for feasibility, risk communication, and implementation, which should be addressed in further research. Further comprehensive modelling studies should evaluate the expected long‐term impact of risk‐based screening on outcomes and costs and support development and implementation of the most effective and cost‐effective risk‐adapted screening strategies.

## AUTHOR CONTRIBUTIONS

**Teresa Seum:** Conceptualization; methodology; formal analysis; visualization; writing – original draft; writing – review and editing. **Marko Mandic:** Writing – review and editing. **Fatemeh Safizadeh:** Writing – review and editing. **Michael Hoffmeister:** Writing – review and editing. **Hermann Brenner:** Conceptualization; methodology; funding acquisition; supervision; writing – review and editing.

## FUNDING INFORMATION

This study was supported by the German Federal Ministry of Education and Research (No. 01KD2104A).

## CONFLICT OF INTEREST STATEMENT

The authors declare no potential conflicts of interest.

## Supporting information


**TABLE S1.** GLOBOCAN Data sources according to Ferlay et al.^25^
**TABLE S2.** Country‐specific 5‐year cumulative risk of colorectal cancer in 2022.
**TABLE S3.** Risk‐adapted starting ages for colorectal cancer screening, by BMI category and country: Sensitivity analysis using people with normal weight (BMI 18.5‐24.9 kg/m^2^) as the reference category.

## Data Availability

The data underlying this article are available from Global Cancer Observatory (GLOBOCAN): Cancer Today (version 1.1). Lyon, France: International Agency for Research on Cancer https://gco.iarc.fr/today/. Further information is available from the corresponding author upon request.

## References

[1] Vuik FER , Nieuwenburg SAV , Bardou M , et al. Increasing incidence of colorectal cancer in young adults in Europe over the last 25 years. Gut. 2019;68(10):1820‐1826. doi:10.1136/gutjnl-2018-317592 31097539 PMC6839794

[2] Lu X‐q , Li Y , Wang W , Feng W‐t , Shi O‐m , Wang Q . International incidence trends in early‐ and late‐onset colorectal cancer: a population‐based study. Int J Color Dis. 2020;35(6):1077‐1086. doi:10.1007/s00384-020-03558-2 32173775

[3] Chang SH , Patel N , Du M , Liang PS . Trends in early‐onset vs late‐onset colorectal cancer incidence by race/ethnicity in the United States cancer statistics database. Clin Gastroenterol Hepatol. 2022;20(6):e1365‐e1377. doi:10.1016/j.cgh.2021.07.035 34325062 PMC8789949

[4] Akimoto N , Ugai T , Zhong R , et al. Rising incidence of early‐onset colorectal cancer ‐ a call to action. Nat Rev Clin Oncol. 2021;18(4):230‐243. doi:10.1038/s41571-020-00445-1 33219329 PMC7994182

[5] Patel SG , Karlitz JJ , Yen T , Lieu CH , Boland CR . The rising tide of early‐onset colorectal cancer: a comprehensive review of epidemiology, clinical features, biology, risk factors, prevention, and early detection. Lancet Gastroenterol Hepatol. 2022;7(3):262‐274. doi:10.1016/S2468-1253(21)00426-X 35090605

[6] Ye P , Xi Y , Huang Z , Xu P . Linking obesity with colorectal cancer: epidemiology and mechanistic insights. Cancer. 2020;12(6):1408. doi:10.3390/cancers12061408 PMC7352519 32486076

[7] Ahrenfeldt J , Carstensen S , Eriksen IMH , Birkbak NJ . Exploring the impact of body mass index on tumor biology and cancer development. J Cancer Res Clin Oncol. 2024;150(7):372. doi:10.1007/s00432-024-05890-4 39068253 PMC11283407

[8] Tang C , Castillon VJ , Waters M , et al. Obesity‐dependent selection of driver mutations in cancer. Nat Genet. 2024;56(11):2318‐2321. doi:10.1038/s41588-024-01969-3 39468367 PMC11549034

[9] Chong B , Kong G , Shankar K , et al. The global syndemic of metabolic diseases in the young adult population: a consortium of trends and projections from the global burden of disease 2000–2019. Metabolism. 2023;141:155402. doi:10.1016/j.metabol.2023.155402 36717058

[10] Phelps NH , Singleton RK , Zhou B , et al. Worldwide trends in underweight and obesity from 1990 to 2022: a pooled analysis of 3663 population‐representative studies with 222 million children, adolescents, and adults. Lancet. 2024;403(10431):1027‐1050. doi:10.1016/S0140-6736(23)02750-2 38432237 PMC7615769

[11] Arnold M , Lam F , Ervik M , Soerjomataram I . Cancer and Obesity: Global burden of cancer attributable to excess weight. International Agency for Research on Cancer. 2025 http://gco.iarc.fr/obesity

[12] Xu P , Tao Z , Yang H , Zhang C . Obesity and early‐onset colorectal cancer risk: emerging clinical evidence and biological mechanisms. Review. Front Oncol. 2024;14:1366544. doi:10.3389/fonc.2024.1366544 38764574 PMC11100318

[13] Davidson KW , Barry MJ , Mangione CM , et al. Screening for colorectal cancer: US preventive services task force recommendation Statement. Jama. 2021;325(19):1965‐1977. doi:10.1001/jama.2021.6238 34003218

[14] Cardoso R , Guo F , Heisser T , Hoffmeister M , Brenner H . Utilisation of colorectal cancer screening tests in European countries by type of screening offer: results from the European health interview survey. Cancers (Basel). 2020;12(6):1409. doi:10.3390/cancers12061409 32486077 PMC7352919

[15] Canadian Task Force on Preventive Health Care . Recommendations on screening for colorectal cancer in primary care. Can Med Assoc J. 2016;188(5):340‐348. doi:10.1503/cmaj.151125 26903355 PMC4786388

[16] NHS England . Bowel cancer screening: programme overview. 2026 https://www.gov.uk/guidance/bowel‐cancer‐screening‐programme‐overview

[17] Dekker E , Tanis PJ , Vleugels JLA , Kasi PM , Wallace MB . Colorectal cancer. Lancet. 2019;394(10207):1467‐1480. doi:10.1016/S0140-6736(19)32319-0 31631858

[18] Shaukat A , Levin TR . Current and future colorectal cancer screening strategies. Nat Rev Gastroenterol Hepatol. 2022;19(8):521‐531. doi:10.1038/s41575-022-00612-y 35505243 PMC9063618

[19] Li H , Boakye D , Chen X , Hoffmeister M , Brenner H . Association of body mass index with risk of early‐onset colorectal cancer: systematic review and meta‐analysis. Am J Gastroenterol. 2021;116(11):2173‐2183. doi:10.14309/ajg.0000000000001393 34309586 PMC8560162

[20] Mandic M , Li H , Safizadeh F , Niedermaier T , Hoffmeister M , Brenner H . Is the association of overweight and obesity with colorectal cancer underestimated? An umbrella review of systematic reviews and meta‐analyses. Eur J Epidemiol. 2023;38(2):135‐144. doi:10.1007/s10654-022-00954-6 36680645 PMC9905196

[21] World Health Organization . Mean Body Mass Index Trends Among Adults, Age‐Standardized (kg/m^2^): Estimates by WHO Region. Global Health Observatory Data Repository. 2025 https://apps.who.int/gho/data/view.main-eu.BMIMEANAREGv?lang=en

[22] Mandic M , Safizadeh F , Niedermaier T , Hoffmeister M , Brenner H . Association of overweight, obesity, and recent weight loss with colorectal cancer risk. JAMA Netw Open. 2023;6(4):e239556. doi:10.1001/jamanetworkopen.2023.9556 37083659 PMC10122181

[23] Hamilton W , Round A , Sharp D , Peters TJ . Clinical features of colorectal cancer before diagnosis: a population‐based case–control study. Br J Cancer. 2005;93(4):399‐405. doi:10.1038/sj.bjc.6602714 16106247 PMC2361578

[24] Wang Q‐L , Babic A , Rosenthal MH , et al. Cancer diagnoses after recent weight loss. Jama. 2024;331(4):318‐328. doi:10.1001/jama.2023.25869 38261044 PMC10807298

[25] Ferlay J , Ervik M , Lam F , et al. Global Cancer Observatory: Cancer Today (version 1.1). Lyon, France: International Agency for Research on Cancer. 2024 https://gco.iarc.who.int/today

[26] McGeoch L , Saunders CL , Griffin SJ , et al. Risk prediction models for colorectal cancer incorporating common genetic variants: a systematic review. Cancer Epidemiol Biomarkers Prev. 2019;28(10):1580‐1593. doi:10.1158/1055-9965.EPI-19-0059 31292139 PMC7610631

[27] Chen X , Li H , Mandic M , Hoffmeister M , Brenner H . Assessment of body mass index, polygenic risk score, and development of colorectal cancer. JAMA Netw Open. 2022;5(12):e2248447. doi:10.1001/jamanetworkopen.2022.48447 36547977 PMC9857417

[28] Usher‐Smith JA , Harshfield A , Saunders CL , et al. External validation of risk prediction models for incident colorectal cancer using UK biobank. Br J Cancer. 2018;118(5):750‐759. doi:10.1038/bjc.2017.463 29381683 PMC5846069

[29] Yeoh A , Mannalithara A , Ladabaum U . Cost‐effectiveness of earlier or more intensive colorectal cancer screening in overweight and obese patients. Clin Gastroenterol Hepatol. 2023;21(2):507‐519. doi:10.1016/j.cgh.2022.07.028 35940514

[30] Genua F , Raghunathan V , Jenab M , Gallagher WM , Hughes DJ . The role of gut barrier dysfunction and microbiome dysbiosis in colorectal cancer development. Front Oncol. 2021;11:626349. doi:10.3389/fonc.2021.626349 33937029 PMC8082020

[31] Janssen F , Bardoutsos A , Vidra N . Obesity prevalence in the Long‐term future in 18 European countries and in the USA. Obes Facts. 2020;13(5):514‐527. doi:10.1159/000511023 33075798 PMC7670332

[32] Rader B , Hazan R , Brownstein JS . Changes in adult obesity trends in the US. JAMA Health Forum. 2024;5(12):e243685. doi:10.1001/jamahealthforum.2024.3685 39671205 PMC11645646

[33] Patel SG , May FP , Anderson JC , et al. Updates on age to start and stop colorectal cancer screening: recommendations from the U.S. multi‐society task force on colorectal cancer. Gastroenterology. 2022;162(1):285‐299. doi:10.1053/j.gastro.2021.10.007 34794816

[34] Siegel RL , Wagle NS , Cercek A , Smith RA , Jemal A . Colorectal cancer statistics, 2023. CA Cancer J Clin. 2023;73(3):233‐254. doi:10.3322/caac.21772 36856579

[35] Graham Y , Hayes C , Cox J , Mahawar K , Fox A , Yemm H . A systematic review of obesity as a barrier to accessing cancer screening services. Obes Sci Pract. 2022;8(6):715‐727. doi:10.1002/osp4.606 36483123 PMC9722456

[36] Telo GH , Friedrich Fontoura L , Avila GO , et al. Obesity bias: how can this underestimated problem affect medical decisions in healthcare? A systematic review. Obes Rev. 2024;25(4):e13696. doi:10.1111/obr.13696 38272850

[37] Safizadeh F , Mandic M , Pulte D , Niedermaier T , Hoffmeister M , Brenner H . The underestimated impact of excess body weight on colorectal cancer risk: evidence from the UK biobank cohort. Br J Cancer. 2023;129(5):829‐837. doi:10.1038/s41416-023-02351-6 37443347 PMC10449928

[38] Safizadeh F , Mandic M , Hoffmeister M , Brenner H . Colorectal cancer and central obesity. JAMA Netw Open. 2025;8(1):e2454753. doi:10.1001/jamanetworkopen.2024.54753 39820694 PMC11739990

[39] Sweatt K , Garvey WT , Martins C . Strengths and limitations of BMI in the diagnosis of obesity: what is the path forward? Curr Obes Rep. 2024;13(3):584‐595. doi:10.1007/s13679-024-00580-1 38958869 PMC11306271

