# Supplementary material for: At what age should people with obesity start colorectal cancer screening?
Source: Int J Cancer. 2026 Apr 9;159(4):876–82. doi: 10.1002/ijc.70448 (PMC13284624; doi:10.1002/ijc.70448)
Supplement: Supplementary file 1 — TABLE S1. GLOBOCAN Data sources according to Ferlay et al. 25 TABLE S2. Country‐specific 5‐year cumulative risk of colorectal cancer in 2022. TABLE S3. Risk‐adapted starting ages for colorectal cancer screening, by BMI category and country: Sensitivity analysis using people with normal weight (BMI 18.5‐24.9 kg/m2) as the reference category. [file IJC-159-876-s001.pdf]

**Supplementary materials**

# At what age should people with obesity start colorectal cancer screening?

*Teresa Seum, Marko Mandic, Fatemeh Safizadeh, Michael Hoffmeister, Hermann Brenner*

***Table of content***

Supplementary Methods ........................................ 2

Supplementary tables ........................................ 6

Supplementary Table 1. GLOBOCAN Data sources according to Ferlay et al. (2024). ........................................ 6

Supplementary Table 2. Country-specific 5-year cumulative risk of colorectal cancer in 2022 according to age ........................................ 7

Supplementary Table 3. Risk-adapted starting ages for colorectal cancer screening, by BMI category and country: Sensitivity analysis using people with normal weight (BMI 18.5–24.9 kg/m$^2$) as the reference category. ........................................ 8

Supplementary references ........................................ 9

**Supplementary Methods**

**Search strategy**

We performed a systematic literature search from inception to January 30th 2025 across PubMed, Web of Science, EMBASE, and the Cochrane Database of Systematic Reviews to identify systematic reviews and meta-analyses assessing the relationship between excess weight, defined as overweight and obesity according to World Health Organisation (WHO) criteria, and the risk of early-onset colorectal cancer (eoCRC) or colorectal cancer (CRC) incidence.

**Eligibility criteria**

Studies were considered eligible if they were systematic reviews or meta-analyses evaluating this association, including site-specific risks for colon and rectal cancer. To be included, they had to conduct a quantitative synthesis, reporting a summary effect estimate along with supporting data from individual studies. When available, we prioritized studies focusing on eoCRC, as lowering screening initiation ages would directly affect individuals under 50. Preference was given to meta-analyses that addressed potential biases, particularly those accounting for pre-diagnostic weight loss, which can lead to an underestimation or reversal of the association between body mass index (BMI) and CRC. As CRC can induce substantial weight loss in its preclinical phase, BMI measurements taken close to diagnosis may not accurately reflect prior excess weight.[13]

Studies were excluded if they were narrative reviews, systematic reviews lacking a quantitative meta-analysis, or if they solely examined CRC mortality. No limitations were imposed regarding publication date or language.

From the identified studies, we selected the most recently published meta-analysis that incorporated the largest number of relevant studies and demonstrated the highest methodological rigor.

**Search Terms**

| |
|---|
| **PubMed** |
| (BMI[Title/Abstract] OR overweight[Title/Abstract] OR "over weight"[Title/Abstract] OR obesity[Title/Abstract] OR obese[Title/Abstract] OR "body mass"[Title/Abstract] OR "body size"[Title/Abstract] OR "body fat"[Title/Abstract] OR "body fatness"[Title/Abstract] OR anthropometr*[Title/Abstract] OR "weight loss"[Title/Abstract] OR "weight gain"[Title/Abstract] OR "body composition"[Title/Abstract] OR "waist circumference"[Title/Abstract] OR "hip circumference"[Title/Abstract] OR "waist hip ratio*"[Title/Abstract])<br><br>AND (("Colorectal Neoplasms"[Mesh] OR "colorectal"[Title/Abstract] OR "colon"[Title/Abstract] OR "rectal"[Title/Abstract] OR "rectum"[Title/Abstract] OR "CRC"[Title/Abstract])<br>AND ("Neoplasms"[Mesh] OR "neoplasms"[Title/Abstract] OR "neoplasm"[Title/Abstract] OR "neoplasia"[Title/Abstract] OR "cancer"[Title/Abstract] OR "tumor"[Title/Abstract] OR "carcinoma"[Title/Abstract]))<br><br>AND ("Meta-Analysis"[ptyp] OR "Systematic Review"[ptyp]) |
| 335 results |
| **SCOPUS** |
| (TITLE-ABS-KEY(BMI OR overweight OR "over weight" OR obesity OR obese OR "body mass" OR "body size" OR "body fat" OR "body fatness" OR anthropometr* OR "weight loss" OR "weight gain" OR "body composition" OR "waist circumference" OR "hip circumference" OR "waist hip ratio*"))<br>AND (TITLE-ABS-KEY("colorectal" OR "colon" OR "rectal" OR "rectum" OR "CRC"))<br>AND (TITLE-ABS-KEY("neoplasms" OR "neoplasm" OR "neoplasia" OR "cancer" OR "tumor" OR "carcinoma"))<br>AND (TITLE-ABS-KEY("meta-analysis" OR "systematic review"))<br>AND ( LIMIT-TO ( DOCTYPE , "re" ) ) |
| 776 results |
| **EMBASE** |
| (bmi:ti,ab OR overweight:ti,ab OR 'over weight':ti,ab OR obesity:ti,ab OR obese:ti,ab OR 'body mass':ti,ab OR 'body size':ti,ab OR 'body fat':ti,ab OR 'body fatness':ti,ab OR anthropometr*:ti,ab OR 'weight loss':ti,ab OR 'weight gain':ti,ab OR 'body composition':ti,ab OR 'waist circumference':ti,ab OR 'hip circumference':ti,ab OR 'waist hip ratio*':ti,ab) AND ('colorectal' OR 'colon'/exp OR 'rectal' OR 'rectum'/exp OR 'crc':ti,ab) AND ('neoplasms'/exp OR 'neoplasm':ti,ab OR 'neoplasia':ti,ab OR 'cancer':ti,ab OR 'tumor':ti,ab OR 'carcinoma':ti,ab) AND ('meta analysis'/exp OR 'systematic review'/exp) |
| 731 results |
| **COCHRANE** |
| BMI OR overweight OR "over weight" OR obesity OR obese OR "body mass" OR "body size" OR "body fat" OR "body fatness" OR anthropometr* OR "weight loss" OR "weight gain" OR "body composition" OR "waist circumference" OR "hip circumference" OR "waist hip ratio*" in Title Abstract Keyword<br>AND "colorectal" OR "colon" OR "rectal" OR "rectum" OR "CRC" in Title Abstract Keyword<br>AND "neoplasms" OR "neoplasm" OR "neoplasia" OR "cancer" OR "tumor" OR "carcinoma" in Title Abstract Keyword |
| 5 results |

**Search results**

Preferred Reporting Items for Systematic Reviews and Meta-Analyses (PRISMA) flow diagram

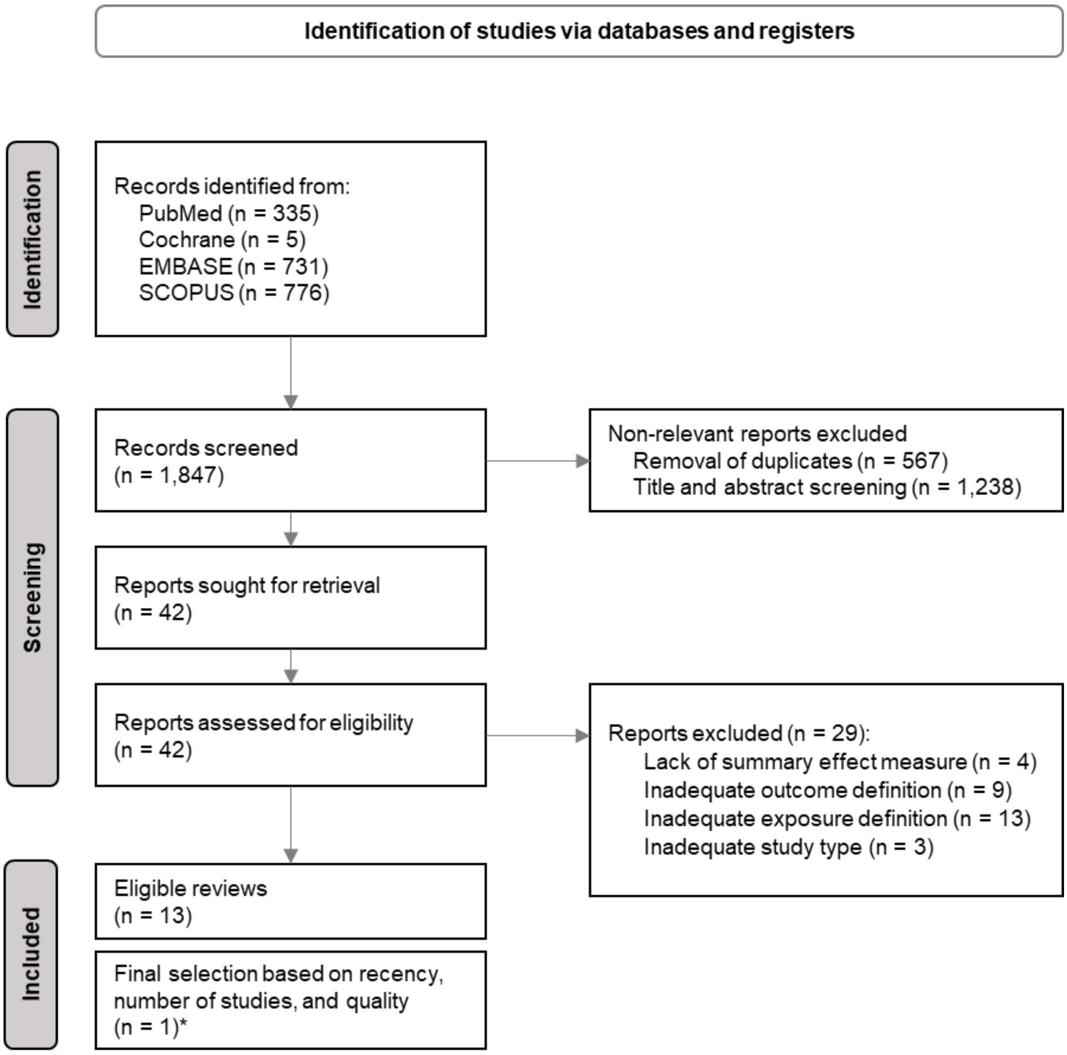


* Li H, Boakye D, Chen X, Hoffmeister M, Brenner H. Association of Body Mass Index With Risk of Early-Onset Colorectal Cancer: Systematic Review and Meta-Analysis. *Am J Gastroenterol*. 2021;116(11):2173-2183. doi:10.14309/ajg.0000000000001393

**Overview of the eligible reviews**

| First author (year) | No. of studies | eoCRC-specific estimates | Addresses pre-diagnostic weight loss |
|---|---|---|---|
| Choi et al. (2018) | 41 on colon cancer, 32 on rectal cancer | No | No |
| Dai et al. (2007) | 15 | No | No |
| Fang et al. (2018) | 34 on colorectal cancer, 35 on colon cancer, 28 on rectal cancer | No | No |
| Freisling et al. (2017) | 7 cohort studies | No | No |
| Harriss et al. (2009) | 29 | No | No |
| Hua et al. (2023) | 36 (10 pooled in meta-analysis on obesity, 7 on overweight) | Yes | No |
| Li et al. (2021) | 13 (6 pooled in meta-analysis) | Yes | Yes |
| Ma et al. (2013) | 41 | No | No |
| Moghaddam et al. (2007) | 31 | No | No |
| Ning et al. (2010) | 56 | No | No |
| O'Sullivan et al. (2022) | 20 (7 pooled in meta-analysis on obesity) | Yes | No |
| Ungvari et al. (2024) | 66 | No | No |
| Zhan et al. (2024) | 12 pooled in meta-analysis of CRC on obesity | No | No |

**Supplementary tables**

**Supplementary Table 1. GLOBOCAN Data sources according to Ferlay et al. (2024).**

| Location | Source | Method | Method Information |
|---|---|---|---|
| **North America** | | | |
| United States of America | National | M1 | Rates (2008-2017) projected to 2022 and applied to 2022 population.<br>Data sources:<br>• CDC, National Program of Cancer Registries: 46 states and the District of Columbia;<br>• NIH, Surveillance, Epidemiology, and End Results (SEER): Connecticut, Hawaii, Iowa and New Mexico |
| Canada | Sub-national | M1 | Rates (all provinces excl. New Brunswick, Newfoundland, Northwest Territories, Nova Scotia and Quebec, 2008-2017) projected to 2022 and applied to 2022 population. |
| **Europe** | | | |
| Germany | Sub-national | M3a | Estimated from mortality using M:I ratios from 8 sub-national cancer registries |
| France (metropolitan) | Sub-national | M3a | Estimated from mortality using M:I ratios from 15 sub-national cancer registries |
| United Kingdom | National | M1 | Pooled England, Northern Ireland, and Scotland rates (2010-2019) projected to 2022 and applied to 2022 population. |
| Italy | Sub-national | M3a | Estimated from mortality using M:I ratios from 36 sub-national cancer registries |

M1 National (or sub-national with coverage greater than 50%) rates projected to 2022; M3a Estimated from national mortality estimates by modelling, using mortality:incidence ratios derived from country-specific cancer registry data

**Supplementary Table 2. Country-specific 5-year cumulative risk of colorectal cancer in 2022 according to age.**

| | 5-year cumulative colorectal cancer risk from age on [%] | | | | | |
|---|---|---|---|---|---|---|
| | **30** | **35** | **40** | **45** | **50** | **55** |
| **North America** | | | | | | |
| United States | 0.04 | 0.08 | 0.14 | 0.22 | 0.34 | 0.43 |
| Canada | 0.03 | 0.06 | 0.11 | 0.18 | 0.27 | 0.38 |
| **Europe** | | | | | | |
| Germany | 0.01 | 0.01 | 0.05 | 0.10 | 0.19 | 0.31 |
| France | 0.02 | 0.04 | 0.09 | 0.16 | 0.26 | 0.41 |
| United Kingdom | 0.02 | 0.03 | 0.07 | 0.13 | 0.24 | 0.40 |
| Italy | 0.01 | 0.03 | 0.07 | 0.13 | 0.22 | 0.36 |

Source: GLOBOCAN 2022 (Ferlay et al., 2024)

**Supplementary Table 3. Risk-adapted starting ages for colorectal cancer screening, by BMI category and country: Sensitivity analysis using people with normal weight (BMI 18.5–24.9 kg/m²) as the reference category.**

| | Risk-adapted starting age [years (95 % confidence interval)] | | | |
|---|---|---|---|---|
| | **Benchmark: Age 45** | | **Benchmark: Age 50** | |
| | **Obesity** (BMI ≥30 kg/m²) | **Overweight** (BMI 25–29.9 kg/m²) | **Obesity** (BMI ≥30 kg/m²) | **Overweight** (BMI 25–29.9 kg/m²) |
| **North America** | | | | |
| United States | 38 (36-41) | 42 (41-43) | 43 (39-46) | 47 (45-48) |
| Canada | 39 (36-41) | 42 (41-43) | 42 (40-46) | 46 (45-48) |
| **Europe** | | | | |
| Germany | 40 (39-42) | 43 (42-43) | 45 (42-47) | 47 (47-48) |
| France | 40 (37-42) | 42 (41-43) | 43 (41-46) | 47 (46-48) |
| United Kingdom | 40 (38-42) | 42 (42-43) | 45 (42-47) | 47 (47-48) |
| Italy | 40 (38-42) | 42 (42-43) | 44 (41-46) | 47 (46-48) |

BMI, body mass index

**Supplementary references**

Choi, E. K., Park, H. B., Lee, K. H., Park, J. H., Eisenhut, M., van der Vliet, H. J., Kim, G., & Shin, J. I. (2018). Body mass index and 20 specific cancers: re-analyses of dose-response meta-analyses of observational studies. *Ann Oncol*, *29*(3), 749-757. https://doi.org/10.1093/annonc/mdx819

Dai, Z., Xu, Y. C., & Niu, L. (2007). Obesity and colorectal cancer risk: a meta-analysis of cohort studies. *World J Gastroenterol*, *13*(31), 4199-4206. https://doi.org/10.3748/wjg.v13.i31.4199

Fang, X., Wei, J., He, X., Lian, J., Han, D., An, P., Zhou, T., Liu, S., Wang, F., & Min, J. (2018). Quantitative association between body mass index and the risk of cancer: A global Meta-analysis of prospective cohort studies. *Int J Cancer*, *143*(7), 1595-1603. https://doi.org/10.1002/ijc.31553

Ferlay, J., Ervik, M., Lam, F., Laversanne, M., Colombet, M., Mery, L., Piñeros, M., Znaor, A., Soerjomataram, I., & Bray, F. (2024). *Global Cancer Observatory: Cancer Today (version 1.1). Lyon, France: International Agency for Research on Cancer*. Retrieved Dec 16 from https://gco.iarc.who.int/today

Freisling, H., Arnold, M., Soerjomataram, I., O'Doherty, M. G., Ordonez-Mena, J. M., Bamia, C., Kampman, E., Leitzmann, M., Romieu, I., Kee, F., Tsilidis, K., Tjonneland, A., Trichopoulou, A., Boffetta, P., Benetou, V., Bueno-de-Mesquita, H. B. A., Huerta, J. M., Brenner, H., Wilsgaard, T., & Jenab, M. (2017). Comparison of general obesity and measures of body fat distribution in older adults in relation to cancer risk: meta-analysis of individual participant data of seven prospective cohorts in Europe. *Br J Cancer*, *116*(11), 1486-1497. https://doi.org/10.1038/bjc.2017.106

Harriss, D. J., Atkinson, G., George, K., Cable, N. T., Reilly, T., Haboubi, N., Zwahlen, M., Egger, M., Renehan, A. G., & group, C. C. (2009). Lifestyle factors and colorectal cancer risk (1): systematic review and meta-analysis of associations with body mass index. *Colorectal Dis*, *11*(6), 547-563. https://doi.org/10.1111/j.1463-1318.2009.01766.x

Hua, H., Jiang, Q., Sun, P., & Xu, X. (2023). Risk factors for early-onset colorectal cancer: systematic review and meta-analysis. *Front Oncol*, *13*, 1132306. https://doi.org/10.3389/fonc.2023.1132306

Li, H., Boakye, D., Chen, X., Hoffmeister, M., & Brenner, H. (2021). Association of Body Mass Index With Risk of Early-Onset Colorectal Cancer: Systematic Review and Meta-Analysis. *Am J Gastroenterol*, *116*(11), 2173-2183. https://doi.org/10.14309/ajg.0000000000001393

Ma, Y., Yang, Y., Wang, F., Zhang, P., Shi, C., Zou, Y., & Qin, H. (2013). Obesity and risk of colorectal cancer: a systematic review of prospective studies. *PLoS ONE*, *8*(1), e53916. https://doi.org/10.1371/journal.pone.0053916

Moghaddam, A. A., Woodward, M., & Huxley, R. (2007). Obesity and risk of colorectal cancer: a meta-analysis of 31 studies with 70,000 events. *Cancer Epidemiol Biomarkers Prev*, *16*(12), 2533-2547. https://doi.org/10.1158/1055-9965.EPI-07-0708

Ning, Y., Wang, L., & Giovannucci, E. L. (2010). A quantitative analysis of body mass index and colorectal cancer: findings from 56 observational studies. *Obes Rev*, *11*(1), 19-30. https://doi.org/10.1111/j.1467-789X.2009.00613.x

O'Sullivan, D. E., Sutherland, R. L., Town, S., Chow, K., Fan, J., Forbes, N., Heitman, S. J., Hilsden, R. J., & Brenner, D. R. (2022). Risk Factors for Early-Onset Colorectal Cancer: A Systematic Review and Meta-analysis. *Clin Gastroenterol Hepatol*, *20*(6), 1229-1240 e1225. https://doi.org/10.1016/j.cgh.2021.01.037

Ungvari, Z., Fekete, M., Varga, P., Lehoczki, A., Fekete, J. T., Ungvari, A., & Gyorffy, B. (2024). Overweight and obesity significantly increase colorectal cancer risk: a meta-analysis of 66 studies revealing a 25-57% elevation in risk. *GeroScience*. https://doi.org/10.1007/s11357-024-01375-x

Zhan, Z. Q., Chen, Y. Z., Huang, Z. M., Luo, Y. H., Zeng, J. J., Wang, Y., Tan, J., Chen, Y. X., & Fang, J. Y. (2024). Metabolic syndrome, its components, and gastrointestinal cancer risk: a meta-analysis of 31 prospective cohorts and Mendelian randomization study. *J Gastroenterol Hepatol*, *39*(4), 630-641. https://doi.org/10.1111/jgh.16477
